# Values‐based cognitive behavioural therapy for the prevention of chronic whiplash associated disorders: A randomized controlled trial

**DOI:** 10.1002/ejp.1945

**Published:** 2022-04-11

**Authors:** Tonny Elmose Andersen, Sophie L. Ravn, Anna Mejldal, Kirsten Kaya Roessler

**Affiliations:** ^1^ Department of Psychology University of Southern Denmark: Syddansk Universitet Odense Denmark; ^2^ Specialized Hospital for Polio and Accident Victims Roedovre Denmark; ^3^ Unit of Clinical Alcohol Research Institute of Clinical Research University of Southern Denmark Odense Denmark

## Abstract

**Background:**

Whiplash is a common traffic‐related injury with up to 50% of those affected continuing to experience symptoms one‐year post‐injury. Unfortunately, treatments have not proven highly effective in preventing and treating chronic symptomatology. The overall aim of this study was to test the effectiveness of an early values‐based cognitive‐behavioural therapeutic intervention (V‐CBT) delivered within 6 months post‐injury in preventing chronic symptomatology compared to wait list controls.

**Methods:**

The study was a two‐armed randomized controlled trial. Participants (*n* = 91) experienced pain, disability and at least one psychological risk factor (e.g. enhanced pain‐catastrophizing) after a whiplash trauma no later than 6 months prior. Participants were randomized to 10 sessions of V‐CBT starting 1 week (group A) or 3 months (group B) post‐randomization. The primary outcome was pain‐related disability, while secondary outcomes were pain intensity, neck‐pain related disability, depression, anxiety, PTSD symptoms, pain‐catastrophizing and kinesiophobia. These were evaluated at baseline and at 3, 6, 9 and 12 months post‐randomization.

**Results:**

At 3 months, group A demonstrated clinically important effects on all outcomes that were significantly better than group B (waitlist). When group B received the intervention at 6 months, they also demonstrated clinically important effects on all outcomes. However, there was a significant difference at 12 months for the primary outcome, in which group B increased their disability levels, while group A remained stable.

**Conclusions:**

While this indicates that an intervention window for early prevention of disability after whiplash injury may exist, this needs to be tested in a truly early intervention.

**Significance:**

An early Values‐based Cognitive Behavioural Therapeutic intervention delivered within 6 months post‐injury (mean days 117) was effective in reducing pain‐related disability and psychological distress compared to the control group that received the intervention later after a three months wait‐list period. The effects were sustained at 12 months follow‐up. The early intervention was significantly more effective in reducing pain‐related disability compared to the control group, indicating that an intervention window for early prevention of disability after whiplash injury may exist.

## 1 INTRODUCTION

Whiplash injury is common with up to 300 new cases annually per 100,000 individuals (Holm et al., 2008, 2011) and is often associated with long‐term pain and disability (Carroll et al., 2009) as well as psychological distress (Craig et al., 2016). The persistent condition lasting beyond three months is defined as whiplash associated disorders (WAD) (Spitzer et al., 1995). Of those with initial symptoms, up to 50% still suffer from chronic WAD one‐year post‐injury (Carroll et al., 2009) and 13% are partly or fully unable to maintain work (Kasch et al., 2011).

Unfortunately, chronic WAD has shown resistance to conservative treatments such as physiotherapy (Michaleff et al., 2014), and recovery mainly happens in the first three to six months post‐injury with only little or no recovery after 6 months (Andersen et al., 2016; Sterling et al., 2010), highlighting the critical nature of this early post‐trauma period and potentially indicating an early intervention window (Kamper et al., 2008). Psychological distress, maladaptive cognitions and pain behaviours within few weeks after the injury may contribute to the development of chronic WAD and disability (Adams et al., 2017; Andersen et al., 2016; Jull et al., 2013; Roelofs et al., 2004). Hence, early targeting of risk factors such as fear‐avoidance behaviours, pain catastrophizing and post‐traumatic stress symptoms may be effective in preventing the development of disability after whiplash injury.

Few studies have applied a preventive intervention focusing on these factors (Adams et al., 2017; Robinson et al., 2013; Sterling et al., 2019; Sullivan et al., 2006). In descriptive studies, Adams et al. (2017) and Sullivan et al. (2006) found that an early psychotherapeutic intervention targeting risk groups with elevated levels of pain catastrophizing and fear‐avoidance beliefs was effective in reducing disability. Moreover, it was indicated that intervention within the first six months was more effective than later intervention (Adams et al., 2017). In a randomized controlled trial, Robinson et al. (2013) found that an exposure‐based intervention targeting feared activities in subacute WAD (<3 months post‐injury) was effective in reducing both pain and disability compared to the control groups. Recently, Sterling et al. (2019) found that an early (<4 weeks post‐injury) physiotherapist‐led stress inoculation training intervention for patients with moderate levels of disability and hyperarousal symptoms combined with guideline‐based exercise was more effective in reducing pain‐related disability compared to guideline‐based exercise alone. Finally, acceptance‐based interventions aiming to increase willingness to engage in everyday life activities in accordance with life values have been found to improved disability in chronic pain and WAD (Hughes et al., 2017; Wicksell et al., 2008). Expanding the fear‐avoidance model (FA model) Vlaeyen et al. (1995), Crombez et al. (2012) underlines how pain‐related disability may not always be about phobic fear of (re)injury and movement. Fear and worry may also be about how pain interferes with important life values. Also, unattainable goals related to pain relief may lead to maladaptive pain behaviours. Hence, helping patients to identify attainable and valuable goals is an important therapeutic process. Insight into the discrepancy between important values and current behaviour is an essential part of working with acceptance and motivation to pursue more realistic and valuable everyday goals.

Hence, the aim of the present study was first to assess whether a values‐based cognitive behavioural therapy programme (V‐CBT) delivered <6 months post‐injury was able to prevent the development of disability and psychological distress after whiplash injury compared to wait list controls. Secondly, the aim was to assess whether the same intervention was effective when delivered after a 3‐month wait list period and thereby investigate whether an early intervention window exists.

## 2 METHODS

### 2.1 Study design

The present study is a two‐group randomized controlled trial. Both interventions groups were randomized within 6 months post‐injury and received the same psychological intervention. However, group A received the intervention within one week of randomization, while group B received it after a wait list period of three months post‐randomization. In addition to baseline assessment before randomization (T0), primary and secondary outcomes were obtained 3 (T1), 6 (T2), 9 (T3) and 12 months (T4) post‐randomization for both groups. At 3 months, group A had received the intervention, while group B had been on a wait list. Between 3 and 6 months, Group B was offered the same intervention. Ongoing treatment for WAD symptomatology in the community, such as physiotherapy and pharmacological treatments, were allowed during the trial for both groups.

### 2.2 Study participants

Participants were considered eligible for inclusion if they were between 18–65 years of age, had sustained a whiplash injury grade I‐II within the last 6 months, and were able to understand Danish. In addition, they had to report an average pain intensity of ≥4 on four 11‐point box scales ranging from 0 (‘No Pain’) to 10 (‘Worst Pain Imaginable’). The four scales assessed the following: pain right now, highest level of pain over the past week, lowest level of pain over the past week and average pain. Moreover, they had to report pain‐related disability corresponding to a score of ≥5 on the Pain Disability Index in at least one of the seven life domains, and elevated levels of distress on at least one of the following psychological risk factors: Pain‐catastrophizing (PCS ≥20), kinesiophobia (TSK ≥39), depression (HADS‐D ≥7), anxiety (HADS‐A ≥7) or post‐traumatic stress symptoms (PTSD‐8 ≥21). Exclusion criteria were treatment for other severe injuries sustained during the whiplash trauma such as fractures or dislocations. Also, neck pain not being the present primary pain problem led to exclusion. Finally, known severe psychiatric disorders or active abuse of alcohol, drugs, or medicine.

### 2.3 Screening procedure

Participants were primarily recruited from the Danish National Patient Register consecutively from November 2013 to August 2017. Individuals who fulfilled the ICD‐10 diagnosis code for a whiplash injury (distorsio columnae cervicalis) and were situated within approximately 1‐h driving distance to the rehabilitation center were identified monthly and contacted by letter with brief information about the trial. A total of 4182 invitation letters were distributed during this period. Interested individuals then sent back contact information, their consent to be contacted by phone and ratings of their pain levels. Participants with pain levels corresponding to an average of 4 or above on the numerical rating scales (0–10) were contacted by phone for a brief telephone interview and additional oral information. A subset of potential participants contacted the research team after advertisements about the trial in clinical practices, at general practitioners, and with an insurance company, and after receiving additional information about the trial, were invited to participate in the same brief telephone interview. Here, the following screening questions were included: Age (18–65 years old); WAD grade I‐II within past six months; no additional severe injuries sustained during trauma; no pre‐existing chronic pain; no severe psychiatric disorder, and no active abuse of alcohol, drugs, or medicine. Estimation of whiplash injury was based on the Danish National Patient Register and self‐report. In addition, a brief qualitative question about pain levels and related disability was included at this point, and it was ensured that they could understand Danish. Individuals who fulfilled criteria and volunteered at this point were invited to a pre‐trial session with one of the study psychologists. These pre‐trial sessions were primarily done by two licensed psychologists (the same two who conducted the intervention), but a minor subset was carried out by psychologist in training. Prior to this, full written information and baseline questionnaires were sent to them by post. At the pre‐trial session, the remaining inclusion criteria were assessed based on the baseline questionnaires. Also, brief oral information was repeated, and any questions were answered. If the individuals at this stage fulfilled all criteria and still were interested in participating, they signed a written consent form and were then randomized.

### 2.4 Randomization and blinding

Participants were randomized according to a computer‐generated randomization schedule of permuted blocks of 4 to 8 carried out by the research team. The randomization schedule was packed in sequentially numbered opaque envelopes. Eligible participants were randomly assigned to either group A or B immediately after the baseline assessment by the research psychologist by drawing the next envelope. Participants in group A were scheduled immediately by the psychologist within the following week, while participants in group B were informed that they would receive a phone call later to arrange their first session after the 3‐month wait list period. The psychologist informed the research team about the randomization results, and a research assistant arranged the first treatment session for group B after the waiting period. Psychologists and participants were not blind to treatment allocation. During the analysis of data and interpretation of results, the study statistician and the research team were blind to treatment allocation. The results were unblinded after final agreement of the analyses and results.

### 2.5 Study setting

All treatment was carried out by two licensed psychologists in a Danish specialized rehabilitation center.

### 2.6 Protocol registration and ethical approval

Ethical approval was obtained from the local science ethical committee (trial number S‐20130103), and a study protocol was published (Andersen et al., 2015). The trial was preregistered at ClinicalTrials.org (ID: NCT02251028). All participants gave written informed consent before study entry, as outlined above, and the treatment was an additional offer to any existing treatment they might receive elsewhere.

### 2.7 Outcome measures

All outcomes were collected at baseline (T0) before randomization and the intervention took place. Furthermore, the same outcomes were collected at 3 (T1), 6 (T2), 9 (T3) and 12‐month (T4) post‐randomization.

#### 2.7.1 Primary outcome

The primary outcome was the Pain Disability Index (PDI) (Pollard, 1984). The PDI measures of how pain interferes with daily life activities on seven separate scales ranging from 0 (‘No Disability’) to 10 (‘Worst Disability’). The scale covers the following seven life domains: Family/home responsibilities, recreation, social activity, occupation, self‐care, sexual behaviour and life‐support activities. The total level of pain‐related disability is calculated by summing all item scores to a total disability score ranging between 0 and 70, with a higher score indicating a higher level of pain‐related disability. The scale has been validated in multiple populations with musculoskeletal pain and showed good internal consistency, test‐retest reliability and construct validity (Soer et al., 2013). In the present study, internal consistency measured by Cronbach´s alpha was for the total scale *α* = 0.81.

#### 2.7.2 Secondary outcomes

Neck pain‐related disability was measured with the Neck Disability Index (NDI) (Vernon & Minor, 1991). The NDI measures how everyday life activities are interfered by neck pain on 10 separate items. Each item has six possible replies, which vary from item to item but are converted to a score from 0 to 5. The total level of neck pain‐related disability is calculated by summing all item scores to a total score between 0 and 50, with a higher score indicating a higher level of neck pain‐related disability. The NDI is one of the most used outcome measures in whiplash. The NDI has recently been validated in a sample of patients with neck pain and showed good internal consistency, test‐retest reliability and construct validity (Young et al., 2019). In the present study, internal consistency measured by Cronbach´s alpha was for the total scale *α* = 0.82.

Pain intensity was measured as the mean score of four 11‐point box scales ranging from 0 (‘No Pain’) to 10 (‘Worst Pain Imaginable’). The use of 11‐point box scales has been recommended for assessing pain intensity (e.g. Ferreira‐Valente et al. (2011); Jensen et al. (1986)). In this study, the four scales assessed the following: pain right now, highest level of pain over the past week, lowest level of pain over the past week and average pain. In the present study, internal consistency measured by Cronbach´s alpha was for the total scale *α* = 0.86.

The Tampa Scale for Kinesiophobia (TSK) was used to measure fear of movement (Miller et al., 1991). The TSK is a scale with 17 items scored from 1 (‘Strongly Disagree’) to 4 (‘Strongly Agree’). Upon inversion of four items, a total score is calculated by summing all item scores; the total score thus ranged from 17 to 68. A higher score indicates a higher level of fear of movement. The TSK has been used in several pain samples and has good internal consistency, construct and predictive validity (Roelofs et al., 2004). In the present study, internal consistency measured by Cronbach´s alpha was for the total scale *α* = 0.78.

Pain catastrophizing was measured according to the Pain Catastrophizing Scale (PCS) (Sullivan et al., 1995), which consists of 13 items covering three subscales of rumination, magnification and helplessness. Each item is scored from 0 (‘Not at all’) to 4 (‘All the time’), thereby adding to a total score between 0 and 52. A higher symptom level indicates more pain‐related catastrophizing. The PCS is widely used in different pain populations including WAD. The Danish version has been validated in both a clinical and a non‐clinical sample and showed good internal consistency as well as construct and predictive validity (Kjøgx et al., 2014). In the present study, internal consistency measured by Cronbach´s alpha was *α* = 0.89.

Symptoms of anxiety and depression were assessed according to the Hospital Anxiety and Depression Scale (HADS) (Zigmod & Snaith, 1983). The scale consists of two separate subscales: One measures symptoms of anxiety, and one measures depressive symptoms. Each subscale consists of seven items with responses ranging from 0 (‘No impairment’) to 3 (‘Maximum impairment’), resulting in a total score for each subscale between 0 and 21. A higher score indicates a higher symptom level. The Danish version of HADS has recently been validated in a large cohort of somatic patients and showed good internal consistency as well as divergent and convergent validity (Christensen et al., 2020). In the present study, internal consistency measured by Cronbach´s alpha was for the total scale *α* = 0.86, for depression *α* = 0.81 and for anxiety *α* = 0.74.

Post‐traumatic stress symptoms were assessed by the PTSD‐8 (Hansen et al., 2010), which is an abbreviated version of the Harvard Trauma Questionnaire part IV. The scale measures the level of posttraumatic stress symptoms on eight items representing the three PTSD symptom clusters according to DSM‐IV‐TR: Avoidance (two items), intrusion (four items), and hyperarousal (two items). Each item is scored on a scale from 1 (‘Not at all’) to 4 (‘Very often’). The responses are summed to a total score between 8 and 32, with a higher score indicating a higher level of post‐traumatic stress symptomatology. The PTSD‐8 has recently been validated against a gold‐standard diagnostic interview in a sample of chronic pain patients. The scale had a good overall accuracy and a good convergent validity as well as good internal consistency (Andersen et al., 2018). In the present study, internal consistency measured by Cronbach´s alpha was for the total scale *α* = 0.82.

### 2.8 Intervention

Both groups received the same manualized V‐CBT intervention programme for the prevention of chronic WAD (Andersen et al., 2015). For an overview of the complete programme, see Table 1.

**TABLE 1:** Content of the values‐based cognitive behavioural therapy program (V‐CBT)

Session and Topic		Aims	Homework/techniques
1–2	Introduction. Affected life domains. Values and life goals.	To introduce the programme. Discuss affected life domains and values based on PDI questionnaire.	Discuss important values with a close relative/friend. Complete values template (important roles and activities).
3	Pain theory and activity engagement according to values.	To introduce pain models and the bio‐psycho‐social perspective. Discuss values and activity engagement. Setting new short‐ and long‐term values‐based goals and actions. Discuss implementing a walking/workout routine (own preference).	Complete week plan according to planned activities. Establish a walking/workout routine.
4	‘Road‐block’ therapy. Negative reinforcement.	To discuss psychosocial barriers for fulfilling goals. Introduce the concept negative reinforcement and the power of habits. Introduce the cognitive ABC model (Activating event, Beliefs, Consequences). Setting new realistic measurable goals.	Work with barriers, cognitive, emotional or practical. Complete week plan according to planned activities. Walking/Workout routine.
5	Unfulfilled expectations. Having a plan B.	To discuss expectations to oneself and how unfulfilled expectations can affect mood and behaviour resulting in maladaptive coping. Learn to work out a plan B.	Complete plan B and set realistic goals for the day. Complete week plan according to planned activities. Walking/Workout routine.
6	The energy balance and psychological barriers for activity engagement. Adjust goals.	Learn to conserve "energy", prioritise in activities. Discuss psychological barriers for activity engagement. Discuss life values and adjust goals accordingly.	Identify energy consuming activities versus activities that increases energy. Use the ABC model. Complete week plan according to planned activities. Walking/Workout routine.
7	Psychological barriers.	To work more in depth with individual psychological distress and barriers for activity engagement, for instance depressive symptoms, PTSD or pain catastrophizing.	Use the ABC model. Complete week plan according to planned activities. Walking/Workout routine.
8	Psychological barriers.	To work more in depth with individual psychological distress and barriers for activity engagement. Work out an exposure hierarchy of feared activities.	In vivo exposure. Complete week plan according to planned activities. Walking/Workout routine.
9	Activity engagement and return to work or relevant activities.	Discuss return to work or relevant activities. Work with fear‐avoidance beliefs and catastrophizing.	In vivo exposure of work‐ related activities. Complete week plan according to planned activities. Walking/Workout routine.
10	Long‐term goals and values.	To discuss progress and values. Set long‐term goals and work out a plan for setbacks and maintenance of progress.	Discuss long‐term goals and plan with a close relative or friend.

Note

Patient workbook and worksheets (danish) for the program can be downloaded at: https://www.researchgate.net/publication/287205358_Treatment_Manual_for_Value‐based_Cognitive_Behavioral_Therapy_Patient_workbook.

The programme consisted of 10 weekly 1‐h sessions of individually delivered V‐CBT. The programme was specifically tailored to prevent the development of persistent disability after whiplash injuries and combined elements from motivational interviewing (Miller & Rollnick, 2013) and acceptance‐based cognitive behavioural therapeutic approaches (Hayes & Smith, 2005; McCracken & Eccleston, 2005). The primary aim of the programme was to reduce pain‐related disability and how pain interfered with the patient's daily life activities. Acknowledging, that pain often negatively influences the ability to engage in everyday activities to the same degree as before the injury, the intervention started by discussing which activities and life‐roles were most affected and most important to the patient. This therapeutic dialogue was initiated by discussing how the patient had rated pain to interfere with daily life activities on each of the seven domains of the PDI. Motivational interview techniques such as open‐ended questions, affirmation and discussion about discrepancies between values and actions were utilized to establish values‐based goals. This assessment and dialogue were the primary focus during the first two sessions and involved a thorough examination of life values and how these were reflected in specific activities, actions, and life‐roles in the patient's everyday life. Supporting this process, the patients were encouraged to discuss important activities and values with a close relative or friend and as homework complete the values template. On the values template the patients were asked to write down important values related to domains such as family life, friends, health, and work and reflect on actions and activities that support or hinder living according to these values. In the following therapeutic dialogue, the most important values were identified and transformed into specific values‐based goals and actions. This was done to ensure that patients remained physically active and stayed engaged in everyday life activities that were important to them. Values‐based actions were supported and evaluated at every session that followed, and psychosocial barriers for achieving these values‐based goals were discussed and problem‐solving strategies applied. Elevated levels of psychological distress, such as pain catastrophizing and fear avoidance beliefs, were targeted by cognitive restructuring and in‐vivo exposure strategy in between sessions. Also, symptoms of depression and anxiety were targeted by cognitive restructuring. Homework was applied in between sessions.

The intervention was carried out by two licensed psychologists with experience in pain rehabilitation and CBT. Both psychologists were trained in the intervention programme and had piloted it prior to commencing the trial. In addition, they received weekly supervision on video recorded sessions for the first three months and as needed throughout the trial period. Handouts and psychoeducative materials were collected in a printed book, which the participants received at the beginning of the intervention.

### 2.9 Statistical analysis

An independent and blinded study statistician undertook the analyses. Sample size was calculated a priori. Based on earlier studies applying CBT and acceptance‐based strategies with functional restoration and activity engagement as their primary goal a moderate effect size was expected (ηp2 = 0.25) and considered to be of clinical importance (McCracken & Eccleston, 2005; Michaleff et al., 2014). Hence, at a 5% level of significance and 80% power it was calculated that there should be 37 patients in each group (A & B). However, to avoid lack of statistical power due to, higher than expected drop‐out at 9 months follow‐up, we decided to account for up to 25% drop out in the sample size calculations. Therefore, to achieve sufficient statistical power 92 participants were randomized. All assessors remained blinded to data and no interim analyses were conducted (Andersen et al., 2015).

The associations between primary and secondary outcomes and interventions were investigated using multilevel mixed‐effects linear models (LMM). These models make it possible to deal efficiently with missing values due to dropout, assuming the dropout mechanism is missing at random (MAR). Thus, all available data were used, and intention‐to‐treat analyses were applied.

Fixed effects included time point (baseline and follow‐up at, 3, 6 and 12 months), group, and a group by time‐point interaction term. Because participants were randomized, the interaction tests for the existence of group‐by‐time‐point interaction. We included a random effect in the model for each subject, allowing each subject to have their individual intercept. The necessity of a random slope was tested and included, as it represented a significant improvement of the model (i.e. the trajectory of outcome could vary randomly per subject). The significance level was set at 5%, and two‐sided analyses were conducted. All analyses were conducted using Stata, version 15.

In terms of missing data, we distinguish between item‐wise missing (some, but not all, items in a scale are missing) and case‐wise missing (all items in a scale are missing/non‐response). Case‐wise missing was addressed using LMM as mentioned above. Incomplete scales with item‐wise missing were assumed to be missing at random (MAR) and were addressed by the multivariate imputation by chained equations (MICE) method of multiple multivariate imputation. We imputed items separately by timepoint (baseline and follow‐ups at 3, 6, 9 and 12 months). Age, sex, non‐missing scale items and treatment group were included as auxiliary variables in the imputation model. A total of 20 imputed datasets were generated and analysed separately, and results were combined using the rules by Rubin (2004). Finally, percentage of patients achieving a Minimal Clinically Important Difference (MCID) on the two disability measures used was calculated for each group at each follow‐up T2‐T4 and risk ratios were calculated (RR = % MCID for group A/B). The MCID for the two disability measures were: for the primary outcome PDI ≥9.5 points (Soer et al., 2012) and for the secondary outcome NDI ≥5 points (Young et al., 2019).

## 3 RESULTS

### 3.1 Protocol deviations

Some deviations from the protocol were made. Due to recruitment difficulties, the inclusion of patients within 3 months post‐injury was extended. Hence the mean/median time since the injury was closer to 4 months (mean/median 117/122 days, SD = 45, range 25–184 days). Also, follow‐up was extended with one additional time point at 12 months post‐randomization to follow the participants throughout one year. Compared to the protocol, a more conservative power analysis allowing for a larger dropout was applied. This was decided early, and no interim analyses were conducted beforehand.

### 3.2 Enrolment and follow‐up

**FIGURE 1:**
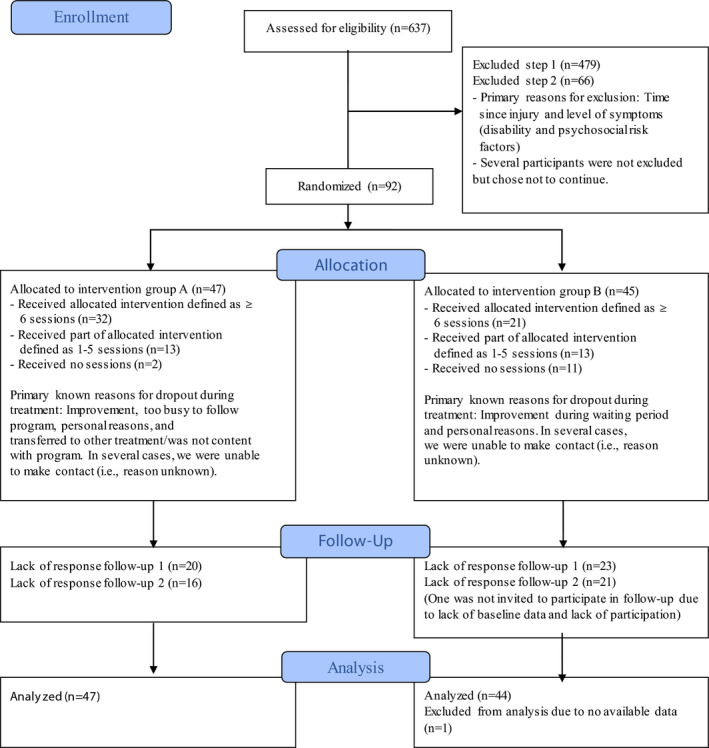
Patient flow

Figure 1 illustrates the patient flow of this study. A total of 637 engaged in the screening procedure, of which 92 fulfilled the inclusion criteria and agreed to participate, but one participant had to be excluded from the analyses due to lack of data. Therefore, 91 patients were included in the final analysis, and intention‐to‐treat analysis was carried out. At both baseline and follow‐ups, there were a few item‐wise missing (i.e. some, but not all, items in a scale are missing). In the TSK, 9% of the cases had up to three items missing at baseline, but at all other timepoints and in all other scales, there were at most 3% (1–2 participants) with item‐wise missing and generally only on 1–2 items. Regarding case‐wise missing, the largest fraction of non‐response was recorded at the follow‐up at 9 months with 47% of the participants not responding. At the follow‐up at 12 months, the case‐wise non‐response was 40%.

### 3.3 Patient compliance

Participants’ treatment compliance was fair, even though some in group B did not start their treatment at all (*n* = 11). The mean number of sessions was 6.1 (standard deviation [SD] = 3.9), and the median was 7 sessions (interquartile range [IQR] = 8). A total of 58.2% received six sessions or more and therefore as outlined in the programme identified important life values and established values‐based goals, 28.6% received one to five sessions, while the remaining 13.2% received no treatment. No adverse events were reported during the trial.

### 3.4 Baseline characteristics and group differences

The mean age was 39.3 years of age (SD = 12.1, range 19–64), and 76.9% were female (*n* = 70). The primary cause of injury was motor vehicle accidents, while a minor part of the sample suffered from whiplash due other incidents such as falls, sport accidents or work‐related accidents. The mean number of days between injury and randomization was 117 (SD = 45) days.

### 3.5 Primary outcome pain disability index

**FIGURE 2:**
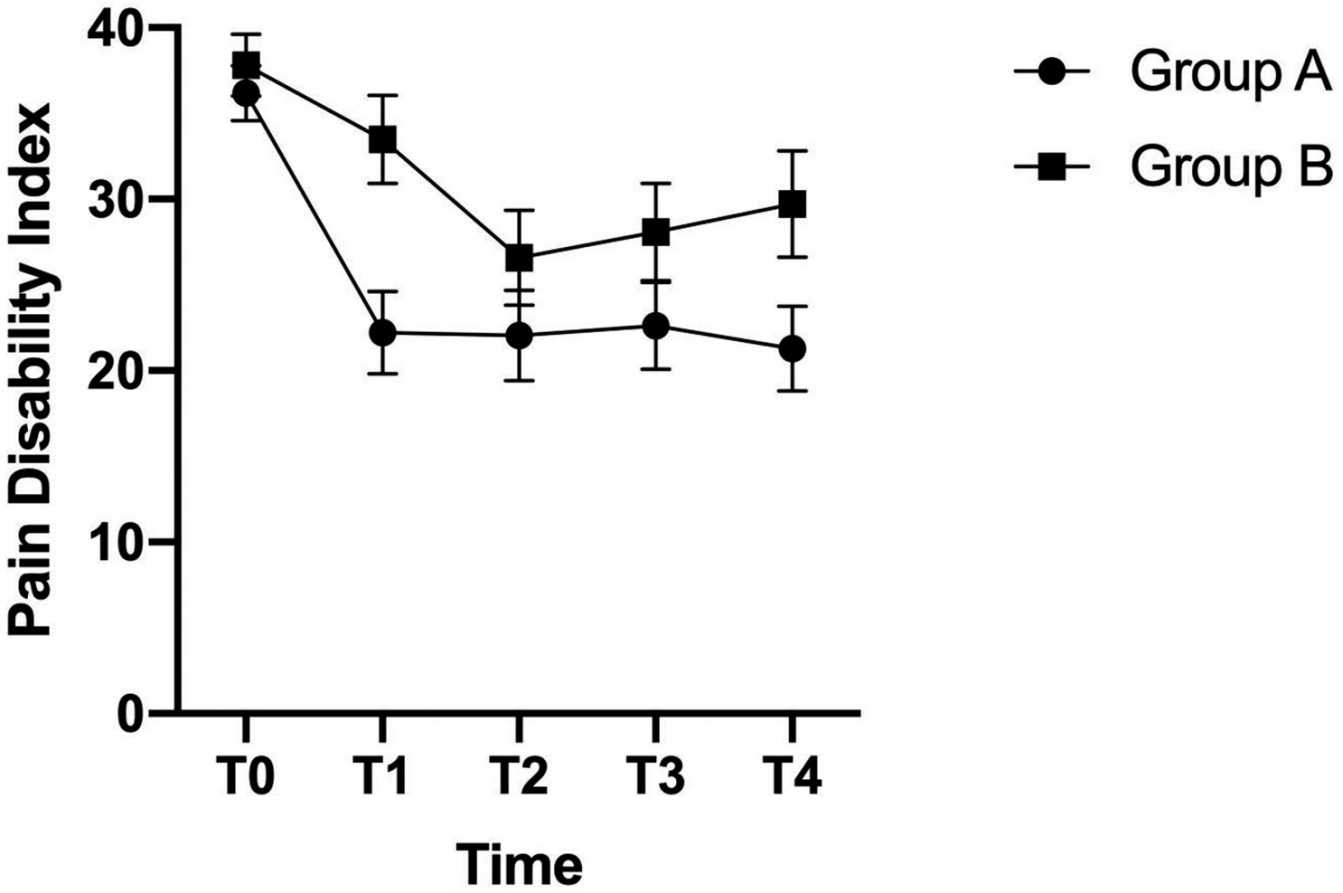
Estimated mean difference and standard error for disability by treatment group and time point. *Note*. Estimated mean difference Pain Disability Index (0–70) with standard error (SE) bars shown. Group A = early intervention, B = late intervention. T1 = 3 months post‐randomization, T2 = 6 months post‐randomization, T3 = 9 months post‐randomization, T4 = 12 months post‐randomization

As shown in Figure 2, the late intervention group (Group B) only had a minimal decline in disability symptoms at the follow‐up at 3 months compared with the early intervention group (Group A) which achieved a large significant decline in disability symptoms. Hence, at 3 months (i.e. before group B had received the intervention), the difference between the two groups was sizable (estimated mean difference PDI = 11.3, SE = 3.5, *p *< 0.01). However, when group B received the intervention (T1‐T2), the participants in this group also achieved a significant decline in disability symptoms.

At the follow‐up at 6 and 9 months (T2‐T3), no significant group differences were found. However, a large and significant difference between the groups was found at the final follow‐up at 12 months (T4) (estimated mean difference PDI = 8.5, SE = 4.0, *p *< 0.05). Overall, group A achieved a decline in disability symptoms of 14.9 points from the baseline to the follow‐up at 12 months, while group B achieved a reduction in disability symptoms of 8.1 points. All treatment effects between the groups at each time point are shown in Table 2.

**TABLE 2:** Treatment effects between group A and B at each time point

Measure	Time	Group A		Group B		Difference (B‐A)	
		Estimate	SE	Estimate	SE	Estimate	SE
PDI (0–70)	T0	36.2	1.6	37.8	1.8	1.6	2.4
	T1	22.2	2.4	33.5	2.6	11.3**	3.5
	T2	22.0	2.6	26.6	2.8	4.5	3.8
	T3	22.6	2.6	28.1	2.8	5.5	3.8
	T4	21.3	2.5	29.7	3.1	8.5*	4.0
NDI (0–50)	T0	22.8	1.1	24.9	1.1	2.2	1.5
	T1	17.0	1.5	23.6	1.3	6.6***	2.0
	T2	17.0	1.3	20.4	1.4	3.4	2.0
	T3	17.0	1.5	20.9	1.8	3.9	2.3
	T4	18.0	1.6	22.4	1.7	4.5	2.3
NRS (0–10)	T0	5.4	0.2	6.0	0.2	0.6*	0.3
	T1	3.1	0.4	5.4	0.3	2.4**	0.5
	T2	3.5	0.4	4.3	0.4	0.8	0.6
	T3	3.5	0.4	4.4	0.5	0.9	0.6
	T4	3.8	0.4	4.6	0.5	0.8	0.6
TSK (17–68)	T0	43.2	0.9	42.4	1.0	−0.8	1.3
	T1	35.0	1.1	39.5	1.1	4.5**	1.6
	T2	35.2	1.0	35.4	1.5	0.2	1.8
	T3	35.6	1.1	35.6	1.6	0.0	1.9
	T4	36.3	1.2	36.2	1.5	−0.1	1.9
PCS (0–52)	T0	25.8	1.4	26.1	1.2	0.3	1.8
	T1	14.4	1.8	23.6	1.6	9.1***	2.4
	T2	13.5	1.8	16.7	2.0	3.2	2.6
	T3	14.0	1.8	17.6	2.2	3.6	2.8
	T4	14.4	1.8	16.0	2.2	1.7	2.8
HADS‐D (0–21)	T0	8.1	0.7	8.8	0.6	0.7	0.9
	T1	5.2	0.7	7.1	0.6	1.9**	0.9
	T2	5.5	0.8	6.1	0.7	0.7	1.0
	T3	5.5	0.8	6.4	0.8	0.9	1.1
	T4	5.7	0.8	7.1	0.9	1.4	1.2
HADS‐A (0–21)	T0	9.9	0.5	10.7	0.6	0.8	0.8
	T1	6.3	0.6	8.6	0.7	2.4**	0.9
	T2	6.5	0.7	6.7	0.7	0.3	1.0
	T3	6.5	0.6	6.7	0.8	0.1	1.0
	T4	6.8	0.8	6.7	0.9	−0.1	1.2
PTSS (8–32)	T0	17.4	0.8	17.0	0.8	−0.4	1.1
	T1	13.2	0.6	16.2	0.7	3.1**	1.0
	T2	13.4	0.7	14.8	1.0	1.4	1.2
	T3	13.8	0.8	15.3	1.0	1.5	1.3
	T4	14.1	0.9	15.3	1.1	1.3	1.4

Note

Group A = early intervention, B = late intervention. T0 = Baseline, T1‐T4 = 3, 6, 9, and 12 months post‐randomization. PDI = Pain Disability Index, NDI =Neck Disability Index, NRS =Pain Intensity Numerical Rating Scale, TSK = TAMPA Scale for Kinesiophobia, PCS = Pain Catastrophizing Scale, HADS‐D = Hospital Anxiety and Depression Scale‐Depression, HADS‐A = Hospital Anxiety and Depression Scale‐Anxiety. PTSS = PTSD‐8.

* *p* = 0.05

** *p* = 0.01

*** *p* = 0.001.

Based on observed data, the percentage of patients that achieved a Minimal Clinical Important Difference on the PDI (MCID ≥9.5; Soer et al., 2012) was for group A versus group B at T1 (74.4.% vs. 31.4%, RR 2.4, *p *< 0.001), T2 (62.5% vs. 52.0%, RR 1.2, *p *> 0.05), T3 (63.0% vs. 44.4%, RR 1.4, *p *> 0.05) and T4 (71.0% vs. 35.0%, RR 2.0, *p* < 0.05).

### 3.6 Secondary outcomes

For all the secondary outcomes, a similar pattern of effects was found; however, the differences were only significant at T1, indicating that the intervention was still effective even though given at a later time point. See Table 2 for group differences and estimates.

Based on observed data, the percentage of patients that achieved a Minimal Clinical Important Difference on the NDI (MCID ≥5; Young et al., 2019) was for group A versus group B at T1 (64.7% vs. 31.6%, RR 2.1, *p *< 0.01), T2 (56.7% vs. 51.9%, RR 1.1, *p* > 0.05), T3 (52.0% vs. 45,0%, RR 1.2, *p* > 0.05) and T4 (57.1% vs. 23.8%, RR 2.4, *p* < 0.05).

## 4 DISCUSSION

### 4.1 Summary of findings

The aim of the present study was firstly to assess whether an early V‐CBT intervention (<6 months post‐injury) was able to prevent the development of disability and psychological distress after whiplash injury compared to wait list controls. Second, the aim was to assess whether the same intervention was still effective when delivered later after a 3‐month wait list period. The early intervention group (A) achieved a significantly larger reduction in pain‐related disability as well as in all secondary outcomes compared to the wait list controls. For the primary outcome, a significantly larger reduction in disability was achieved for the early intervention group at 12 months follow‐up compared to the late intervention group. However, the late intervention proved equally effective as the early intervention on the secondary outcomes.

### 4.2 Accordance with existing literature

The results are in accordance with earlier non‐randomized findings, indicating that early exposure therapy for feared activities may be effective in reducing both pain and disability (Miller & Rollnick, 2013; Soer et al., 2013). The finding that pain‐related disability at the follow‐up at 12 months was significantly smaller in the early intervention group compared to the late intervention group indicates that pain behaviours may be established early (within the first 6 months) after the injury. Furthermore, when pain behaviours are consolidated, they may be more difficult to reverse than if targeted early after the injury. It is still unknown whether an even earlier intervention would have further prevented the development of disability as indicated in the non‐randomised study by Adams et al. (2017).

The present finding that the later intervention was almost as effective as the early intervention in reducing psychological risk factors such as pain‐catastrophizing, fear‐avoidance beliefs, anxiety and depression indicates, as hypothesized by Adams et al. (2017), that the combination of not only activity scheduling and graded activity, but also values‐based CBT techniques is important in order to reduce psychological distress and negative cognitions. The current finding that an early psychological intervention for high‐risk patients was effective in reducing pain‐related disability is opposite of Jull et al. (2013), who found no additional effects of multidisciplinary individualized treatment for acute WAD. The reason for the divergent finding may be explained by the targeting of high‐risk patients based on known risk factors such as pain‐catastrophizing, fear‐avoidance beliefs, PTSS, anxiety and depression, which happened in the present study, while this was not the case in the trial by Jull et al. (2013). The reduction in both pain intensity (NRS) and pain‐related‐disability (PDI and NDI) is interesting, since no exercise‐based intervention was given. Whether an addition of physiotherapy exercises could have strengthened the results is unknown; however, earlier studies find that physiotherapy exercises alone are not very effective in preventing pain‐related disability after WAD, at least not when the condition has become chronic Rubin (2004).

### 4.3 Clinical implications

The present study has important clinical implications as it demonstrates that early psychological care (<6 months post‐injury) for WAD is able to diminish the development of disability and distress for a subgroup of patients presenting with high levels of disability, pain, and distress at baseline; a group that has shown to be at high risk of remaining disabled one year after the injury (Roelofs et al., 2004). These treatment principles can be included in the work of other clinical groups, such as physiotherapists, which should be further investigated in order to improve access to such treatment programmes for WAD patients. Furthermore, a future study should aim to target high‐risk patients even earlier than was possible in the present study, since the present findings indicate that early intervention may be more effective in preventing chronic WAD.

### 4.4 Limitations

The present study has a number of limitations that need to be mentioned. Most importantly, although data were found to be missing at random, even the best imputation methods and intention‐to‐treat analysis are still limited by the high levels of missing values at the follow‐ups at 9 and 12 months. In addition, a number of protocol deviations were made, as outlined in the result section. Extending the inclusion criteria to include patients up to 6 months post‐injury do unfortunately make it difficult to examine the potential existence of a true early intervention window for the prevention of chronic WAD. Although time since injury varied and therefore could be assessed as a potential effect modifier, this was not a preregistered aim, and the study was not statistically powered for this analysis. Moreover, time since injury did not correlate significantly with outcome at any timepoint. However, at 12 months follow‐up the early intervention group was significantly less disabled compared to the late intervention group, indicating that early intervention may be more effective. Also, the choice of design compromises the ability to retain a wait list control group for the whole follow‐up period. Hence, a three‐armed intervention design with a control group for the whole period would have strengthened the study.

While it is a strength that known risk‐factors were used as inclusion criteria and targeted by the intervention, it is still not known which where the active mechanisms of change. The intervention applied a broad range of intervention strategies, spanning from cognitive restructuring to acceptance‐based approaches utilizing values‐based goal setting and activity engagement. More knowledge about specific mechanisms of change can potentially make the intervention more effective. Interpretation of the results could be strengthened if a more structured registration of specific sessions and homework completed was used. Also, the intervention was delivered by licensed psychologists, which may be a limitation in relation to early intervention. A psychological intervention is seldom the first choice of treatment and demands an early screening for high‐risk patients to be effective. A biomedical understanding of the injury may restrain both patients and health practitioners from choosing a psychological intervention as their first choice of treatment. Hence, standard referral procedures and health care expectancies needs to be challenged before early psychological interventions can be implemented into practice. Furthermore, testing the efficacy of the intervention if delivered by other health professionals is warranted. Finally, although large reductions in pain‐related disability was achieved by the early intervention, patients remained moderately disabled. A combination of the early psychological interventions with an early exercise programme may increase the effect and early access to patients.

## 5 CONCLUSIONS

The V‐CBT intervention was effective in reducing both primary and secondary outcomes compared to the wait list controls. Most of the effects were sustained at the follow‐up at 12 months. Moreover, the early intervention was significantly more effective in reducing pain‐related disability comparing the intervention groups at 12 months post‐randomization, indicating that an intervention window for early prevention of disability after whiplash injury may exist. However, this group difference was not found for most of the secondary outcomes. A future study should assess mechanisms of change and whether the intervention is feasible in primary care delivered by physiotherapists.

## CONFLICTS OF INTEREST

None declared.

## AUTHOR CONTRIBUTION

All authors contributed to the concept and design or analysis and interpretation of the results. Also, all authors discussed the results and commented on the manuscript throughout the writing process and approved the final version. The first and second author drafted the manuscript. The third author did all the statistical analyses.
